# High awareness but low utilization: How perceived institutional support shapes college students’ mental health through stigma?

**DOI:** 10.3389/fpsyg.2026.1897669

**Published:** 2026-09-17

**Authors:** Deyun Luo, Xinchun Gu, Huizhu Wang, Ling Zhang

**Affiliations:** 1 School of Digital Commerce, Nanjing Vocational College of Information Technology, Nanjing, China; 2 School of Trade Finance, Wuxi City College of Vocational Technology, Wuxi, China; 3 State Key Laboratory of Water Pollution Control & Green Resource Recycling, School of Environment, Nanjing University, Nanjing, China; 4 College of Economics and Management, Nanjing Agricultural University, Nanjing, China

**Keywords:** mental health, perceived stigma, psychological counseling services, university mental health support, university students

## Abstract

In the context of high awareness but low utilization of university counseling services, an important question is whether university mental health support may still carry psychological significance when actual service use remains limited. Drawing on social support theory and stigma theory, this study extends the understanding of university mental health support beyond a utilization-based perspective by examining whether students’ perceptions of university mental health support are associated with mental health through perceived stigma. Using survey data from 642 university students in China, this study developed an analytical model examining the associations among perceived institutional support, perceived stigma, and mental health outcomes. The results showed that perceived institutional support was positively associated with mental health and negatively associated with perceived stigma. In addition, perceived stigma was negatively associated with mental health and statistically accounted for part of the observed association between perceived institutional support and mental health, suggesting an indirect association consistent with the proposed theoretical model. These findings suggest that the potential value of university mental health support may extend beyond actual counseling utilization. Rather, students’ perceptions of institutional recognition, accessibility, and acceptance of psychological support may represent an important aspect of the campus mental health environment. This perspective is particularly relevant within the Chinese higher education context, where concerns about social evaluation and stigma may influence students’ willingness to acknowledge psychological difficulties and seek support. This study contributes to the literature by moving beyond a utilization-based framework and highlighting the potential contextual significance of perceived institutional support in relation to stigma and mental health. Practically, the findings suggest that Chinese universities may consider not only service availability but also students’ perceptions of institutional support when evaluating campus mental health environments.

## 1 Introduction

University students worldwide are increasingly facing significant mental health challenges. Elevated levels of depression, anxiety, and stress have been widely documented, underscoring the growing urgency of this public health issue (Li et al., 2022; Ahmed et al., 2023; Paiva et al., 2025).

Empirical evidence consistently indicates that mental health problems are prevalent among university students in China (Lin et al., 2025; Gao et al., 2020). Our survey reveals that approximately 12% of university students score within the at-risk range (below 3 on a 5-point scale), suggesting a certain degree of group pervasiveness in psychological risks. In practice, Chinese universities have established institutionalized systems for psychological intervention and support. These systems are typically administered by university-level departments and staffed by professionals, including counseling specialists and student affairs staff, forming an important component of the university’s mental health support infrastructure.

However, a substantial disconnect exists between the availability of university’s mental health support and its actual utilization. Our data indicate that although 92% of students reported being aware of university psychological counseling services and 78% reported knowing how to access these services when needed, only 7% had actually utilized them. This pattern is consistent with findings from the 2024 Mental Health Survey Report of Chinese College Students, which reported a high awareness rate (80%) but a substantially lower utilization rate (2.8%) of psychological counseling services (Fang et al., 2025). These findings suggest that low utilization cannot be fully attributed to insufficient awareness or limited access to information alone, highlighting the importance of examining additional psychological and social factors that associated with students’ willingness to seek professional support.

This high-awareness but low-utilization paradox suggests a substantial gap between the provision of university’s mental health support and its practical uptake, highlighting the need to reconsider how the effectiveness of counseling services is assessed. This paradox leads to the central research question of this study: Does the university’s mental health support system remain functional when the vast majority of students do not actively use it? If only a small minority enter the counseling room, can the remaining majority still benefit from the system’s existence? In other words, does low utilization necessarily equate to the failure of university’s mental health support?

Existing research has largely adopted a functional perspective, treating counseling as an intervention whose effectiveness depends on actual use and is evaluated based on post-treatment outcomes (Ebert et al., 2017; Cuijpers et al., 2018; Lipson et al., 2019). While this approach provides important insights into the benefits of professional services, it offers limited explanatory power for the awareness–utilization gap observed in practice. This study argues for a shift toward a cognitive and contextual perspective to re-examine university’s mental health support, viewing it as a vital institutional contextual cue that functions by reshaping students’ perceptions and evaluations of mental health issues. As an institutional arrangement, the presence of psychological support conveys a normative signal through its availability and authoritative endorsement that psychological distress is a legitimate concern rather than a personal failing and that help-seeking is socially sanctioned (Link and Phelan, 2001). This destigmatizing signal helps improve the socio-cognitive environment at a macro level, fostering a sense of psychological safety across the student body.

Most empirical studies have followed an intervention–benefit path, conceptualizing the effectiveness of counseling services primarily in terms of their direct clinical outcomes for help-seekers, typically reflected in reductions in depressive and anxiety symptoms, alongside improvements in psychological well-being and help-seeking attitudes (Fu et al., 2020; Andersson et al., 2019; Gulliver et al., 2010). Consequently, relatively little attention has been paid to the broader student population who do not actively seek help. In addition, the indirect and symbolic role of university mental health support has remained largely underexplored (Lipson et al., 2019; Osborn et al., 2022). Although stigma is widely recognized as a major barrier to help-seeking (Lannin et al., 2016; Vogel et al., 2007), empirical research remains limited regarding how university mental health support is associated with stigma perceptions and students’ overall mental health. Existing studies have rarely examined university mental health support as a potential institutional resource related to stigma reduction and mental health outcomes (Hatzenbuehler, 2014; Deng et al., 2025; Garcia et al., 2025; AlSamhori et al., 2024).

Against this backdrop, this study develops an analytical framework to explore whether and how university mental health support is associated with students’ mental health outcomes through its relationship with perceived stigma. By controlling for perceived support from family or/and friends, we aim to isolate and clarify the unique signaling value of university’s mental health support within the broader social support network. This analysis not only helps resolve the high awareness low utilization paradox but also highlights the foundational role of university’s mental health support in cultivating a psychologically safe campus culture.

This study makes two contributions by offering a new perspective and exploring the potential relationships among university mental health support, perceived stigma, and mental health. First, by shifting from a conventional utilization-based orientation to a cognitive-signaling framework, this research highlights the symbolic value and potential role of university’s mental health support in shaping students’ perceived stigma and mental health. This perspective provides new insights into the coexistence of high awareness of university’s mental health support resources and relatively low utilization rates. Second, the study explores a potential pathway through which university’s mental health support presence, as a form of institutional endorsement, is associated with students’ perceived stigma and mental health outcomes. By extending the application of social support theory to the organizational context of higher education, this study contributes to a deeper understanding of how students’ perceptions of university mental health support systems are associated with their mental health outcomes.

## 2 Theory and hypotheses

### 2.1 Social support theory and mental health

Social support theory provides an analytical framework for understanding the relationship between university mental health support resources and individual mental health. Sarason et al. (1983) conceptualized social support as a relational resource that may exist both objectively and subjectively, through which individuals experience care, acceptance, and a sense of being valued. Building on this perspective, social support has long been recognized as a protective factor for mental health, as it can exert both direct beneficial effects and buffer the adverse effects of stress (Cohen and Wills, 1985; House et al., 1988; Thoits, 2011).

Supportive resources may provide emotional comfort, informational guidance, and a sense of belonging, thereby alleviating psychological distress and enhancing individuals’ coping capacity. A substantial body of empirical research has shown that higher levels of support are associated with lower levels of depression, anxiety, and psychological distress (Grey et al., 2020; Hefner and Eisenberg, 2009; Vicary et al., 2025). This protective role is particularly salient among students in higher education, who often face multiple academic, developmental, and social stressors (Hefner and Eisenberg, 2009; Beiter et al., 2015).

In this study, we introduce the concept of institutional support to capture university-based mental health support resources and services provided through formal organizational structures and institutional arrangements. These resources include university counseling centers and support provided by faculty members and student affairs counselors in their official roles. Unlike non-institutional support derived from personal relationships with family members and friends, institutional support is characterized by professional expertise, standardized service provision, and formal institutional commitment.

The core logic of this study lies in the distinction between actual institutional support utilization and perceived institutional support. As noted in the introduction, university mental health services often face a paradox of high awareness but low utilization, suggesting that the value of institutional support may not depend entirely on an individual’s actual help-seeking behavior. Therefore, this study focuses on the relationship between students’ perceived institutional support and their mental health.

Within the framework of social support theory, students’ perceived institutional support is viewed as an important dimension of social support that is closely related to mental health outcomes, beyond individuals’ actual utilization of support resources (Wills and Shinar, 2000). Perceived institutional support reflects students’ subjective evaluations of the availability, accessibility, and effectiveness of university-based mental health support resources.

When students perceive that the university has established a comprehensive and accessible mental health support system, this perception itself acts as a positive institutional contextual cue, even if they never physically enter a counseling room. It conveys a signal of organizational care and support, thereby enhancing an individual’s sense of security and psychological resilience. Consequently, even in a context of low utilization, institutional support may still be associated with better mental health outcomes through students’ psychological expectations and cognitive evaluations of available support resources. Accordingly, this study proposes the following hypothesis:

*H1*: Students’ perceived institutional support are positively associated with their mental health.

### 2.2 Stigma theory and mental health

While social support theory highlights the protective role of institutional support, stigma theory helps explain why the availability of institutional support does not always translate into psychological benefits. Stigma refers to socially constructed stereotypes, prejudices, and negative evaluations directed toward individuals whose characteristics are perceived as deviating from prevailing social norms (Goffman, 1963; Link and Phelan, 2001). In the context of mental health, stigma often manifests in beliefs that individuals experiencing psychological difficulties are weak, incompetent, or personally responsible for their problems (Clement et al., 2015; Kotera et al., 2022).

Beyond public stigma, individuals may internalize these negative beliefs, leading to shame, self-blame, and diminished self-worth. A growing body of research has demonstrated that internalized stigma is associated with increased psychological distress, reduced self-efficacy, and poorer mental health (Corrigan et al., 2006; Livingston and Boyd, 2010; Ritsher et al., 2003). Importantly, stigma not only influences help-seeking behavior but also shapes individuals’ self-evaluation and perceptions of social judgment, thereby directly affecting mental health outcomes (Corrigan et al., 2009; Clement et al., 2015).

For students in higher education, concerns about being negatively evaluated or socially labeled may be particularly salient due to the evaluative nature of academic and social environments. Such concerns may intensify psychological burden, inhibit emotional expression, and undermine the effectiveness of available support resources.

*H2*: Students’ perceived institutional support is negatively associated with perceived stigma.

### 2.3 The potential role of perceived stigma

Integrating social support theory and stigma theory, this study conceptualizes perceived stigma as a potential psychological factor associated with the relationship between students’ perceived institutional and mental health outcomes. This framework moves beyond traditional functionalist perspectives by highlighting that institutional support may carry cognitive and normative significance beyond its direct service functions.

Specifically, students’ perceived institutional support functions as an institutional signal (Connelly et al., 2011) and reflects individuals’ perceptions of organizational care and legitimacy (Eisenberger et al., 1986). When students perceive that the college has established a professional and legitimate mental health support system, this system itself serves as a powerful environmental cue that shapes collective norms. Such signals communicate that psychological distress is not a moral flaw or a deficit in capability, but a common life experience, and that seeking professional help is a legitimate and institutionally endorsed behavior. Consistent with stigma theory, normative endorsement from institutions can counteract negative social evaluations and reduce individuals’ stigmatized perceptions of mental health issues (Hatzenbuehler, 2016).

It is important to note that this potential de-stigmatizing role does not necessarily depend on actual service utilization. Even when students have not engaged in help-seeking behaviors, the presence of institutional support resources may still be associated with perceptions of psychological safety and better mental health outcomes. Based on the logic above, the following hypothesis is proposed:

*H3*: Perceived stigma is involved in the association between perceived institutional support and mental health.

## 3 Method

### 3.1 Participants and procedure

This study employed a cross-sectional survey design to examine the relationships among perceived institutional support, perceived stigma, and mental health among Chinese university students. Due to the absence of a comprehensive sampling frame covering all university students in China, a non-probability snowball sampling approach was adopted. Data were collected through an anonymous online questionnaire administered via the Wenjuanxing platform between December 17 and December 27, 2025.

Initial participants were recruited from several public higher education institutions in Jiangsu Province through university networks, personal contacts, and student organizations. After completing the survey, participants were invited to share the questionnaire link with other eligible college students. To increase sample heterogeneity, recruitment involved students from different types of higher education institutions, including general universities and vocational colleges. Ultimately, participants were recruited from multiple public higher education institutions located in seven provinces in China, although the majority of participants were from Jiangsu Province. The recruitment process aimed to include students with diverse demographic and educational backgrounds, including variation in gender, academic year, and institution type.

To protect the confidentiality of participating institutions, the names of the universities are anonymized and presented using numerical identifiers (e.g., University 1, University 2). Detailed information regarding participating institutions and the number of recruited students from each institution is provided in Table 1.

**Table 1:** Distribution of participants across universities.

Province	Institution name	Institution type	Number
Jiangxi	University 1	University	11
Beijing	University 2	University	11
	University 3	University	12
	University 4	University	11
Jiangsu	University 5	University	14
	University 6	Vocational college	60
	University 7	University	12
	University 8	Vocational college	161
	University 9	University	13
	University 10	University	7
	University 11	Vocational college	28
	University 12	University	10
	University 13	Vocational college	19
Anhui	University 14	University	10
	University 15	University	9
	University 16	University	10
	University 17	University	10
	University 18	University	12
	University 19	University	13
	University 20	University	10
	University 21	University	11
	University 22	University	10
	University 23	University	12
	University 24	University	12
	University 25	University	15
	University 26	University	15
	University 27	Vocational college	8
	University 28	University	39
	University 29	University	9
Yunnan	University 30	University	10
Shandong	University 31	University	18
	University 32	Vocational college	10
Sichuan	University 33	University	10
	University 34	University	10
	University 35	University	10
Total			642

University categories were classified according to the official classification of higher education institutions in China. Data were collected from the authors’ survey.

A total of 713 questionnaires were initially collected. Participants were excluded according to predefined data-quality criteria if they completed the questionnaire in an unrealistically short time (i.e., <3 min), demonstrated non-sincere responding patterns (e.g., straight-line responses), had more than 20% missing items, or provided logically inconsistent responses. After data screening, 642 valid questionnaires were retained for analysis, representing 90.04% of the collected questionnaires.

The required sample size was evaluated using mediation-specific power analysis. Following Fritz and MacKinnon (2007), sample size requirements for detecting indirect effects were evaluated based on the expected magnitude of the component paths (path a and path b). Given the observed small-to-moderate association for path a and moderate-to-large association for path b, the required sample size was estimated to be approximately 263 participants to achieve 80% power at an alpha level of 0.05. The final sample size of 642 valid responses therefore provided sufficient statistical power to detect the hypothesized indirect association.

### 3.2 Variable measurement

#### 3.2.1 Perceived institutional support

In this study, perceived institutional support refers to the extent to which students perceive their university as recognizing, endorsing, and providing accessible and supportive mental health resources and services. It specifically reflects support at the university level rather than support provided by individual academic units, healthcare organizations, or other external institutions. Rather than assessing actual help-seeking behaviors or the objective availability of support resources, this study focuses on students’ subjective perceptions of institutional support, including their evaluations of the accessibility, effectiveness, and emotional value of available mental health resources and services.

Perceived institutional support was measured using a newly developed scale based on the theoretical framework of social support and the research objective of examining students’ perceptions of university-based psychological support systems. The conceptual foundation was informed by the Social Support Rating Scale (SSRS; Xiao, 1994), one of the most widely used measures of social support in Chinese populations, which distinguishes among subjective support, objective support, and support utilization.

However, the present scale was not a direct adaptation or translation of the SSRS. Instead, three items were developed to capture students’ perceptions of psychological support provided by institutional and professional sources, including college psychological counselors, academic advisors, and teachers. A sample item is: “When I encounter psychological distress, professional counselors at my college are willing to help me.” All items were rated on a 5-point Likert scale ranging from 1 (strongly disagree) to 5 (strongly agree).

The psychometric properties of the scale were rigorously tested, demonstrating satisfactory reliability and preliminary evidence of construct validity. Specifically, the Cronbach’s α for the three-item scale was 0.878, with item-rest correlations ranging from 0.689 to 0.816, indicating strong internal consistency. An Exploratory Factor Analysis (EFA) supported a unidimensional structure, with a single factor extracted (eigenvalue = 2.05) and factor loadings ranging from 0.727 to 0.879. In addition, bivariate correlations among the main study variables were consistent with theoretical expectations, providing further support for the scale’s validity. For empirical analysis, mean scores were computed, with higher scores reflecting higher levels of perceived institutional support.

#### 3.2.2 Perceived stigma

In this study, perceived stigma was measured using an adapted scale developed based on existing instruments assessing self-stigma and public stigma related to psychological help-seeking. The original items were translated and adapted to the Chinese university context through expert review and pilot testing. Self-stigma items were adapted from the Self-Stigma of Seeking Help Scale (SSOSH) (Vogel et al., 2006) to assess the extent to which seeking professional help threatens an individual’s self-worth. Public stigma items were adapted from the Stigma Scale for Receiving Psychological Help (SSRPH) (Komiya et al., 2000), which captures the perceived degree of devaluation or negative evaluation from others toward those seeking professional support. These two instruments reflect two closely related and theoretically meaningful dimensions; prior research consistently treats self-stigma and public stigma as distinct yet interconnected components of the help-seeking process (Vogel et al., 2007).

Although the original combined scale consisted of 15 items (10 for self-stigma and 5 for public stigma), an item analysis was conducted to optimize the measurement structure. Three items from the self-stigma subscale (Self2, Self5, and Self6) were excluded because their corrected item-total correlations were below the recommended threshold of 0.30. Specifically, the corrected item-total correlations of Self2, Self5, and Self6 were −0.033, 0.258, and 0.135, respectively, indicating insufficient consistency with the overall construct (see Table S1). After removing these items, the final scale consisted of 12 items. Cronbach’s α increased from 0.853 to 0.896, indicating satisfactory internal consistency.

To further evaluate the construct validity of the revised scale, confirmatory factor analysis (CFA) was conducted based on the theoretically proposed two-dimensional structure of perceived stigma. The two-factor correlated model demonstrated an acceptable model fit (χ^2^/df = 2.39, CFI = 0.920, TLI = 0.908, RMSEA = 0.046, SRMR = 0.078). All retained items showed statistically significant and satisfactory standardized factor loadings (all >0.50), providing evidence for the adequacy of the measurement model and the convergent validity of the revised scale.

All items were rated on a 5-point Likert scale (1 = strongly disagree, 5 = strongly agree), with positively worded items in the self-stigma subscale reverse-coded prior to analysis. Sample items include: “If I sought help from a psychotherapist because of psychological problems, I would feel inadequate” (self-stigma) and “Seeking professional psychological help is a sign of personal weakness or incompetence” (public stigma). An overall index of perceived stigma was formed by calculating the mean score, with higher scores indicating stronger stigma.

#### 3.2.3 Mental health

In this study, mental health refers to subjective psychological well-being and emotional functioning rather than clinically diagnosed mental disorders. It was assessed using the Mental Health (MH) subscale of the Chinese version of the 36-Item Short Form Health Survey (SF-36) (Ware and Sherbourne, 1992). The Chinese mainland version of the SF-36 has undergone systematic translation, cultural adaptation, psychometric validation, and normalization procedures (Ren et al., 1998; Li et al., 2003). The MH subscale consists of five items assessing psychological well-being and negative emotional states over the past 4 weeks, including nervousness, low mood, calmness, and happiness. The validated Chinese version was directly adopted in the present study without further modification. Following the scoring procedure of the original SF-36, higher MH scores indicate better perceived mental health.

The five items cover both negative and positive aspects of mental health, including nervousness, depressed mood, calmness, and happiness. Sample items include: “Have you felt very nervous?,” “Have you felt so down in the dumps that nothing could cheer you up?,” and “Have you felt calm and peaceful?.” Participants rated the frequency of each feeling on a 5-point Likert scale. Negatively worded items were reverse-coded so that higher scores reflect better mental health. Item scores were then averaged to generate a composite mental health index. In this study, the scale demonstrated good internal consistency, with a Cronbach’s α of 0.76.

#### 3.2.4 Questionnaire development and pilot testing

Before data collection, the questionnaire was reviewed by 2 doctoral researchers in sociology to evaluate conceptual clarity, cultural appropriateness, and the suitability of item wording. In addition, a pilot test was conducted with 15 university students to assess whether respondents could accurately understand the meaning of each item and complete the questionnaire as intended. Minor revisions were made based on their feedback to improve clarity and readability.

### 3.3 Data analysis

Data analysis was primarily conducted using Stata 18.0. The analytical procedure consisted of three main stages:

First, preliminary analyses were conducted to explore the data distribution and associations between key variables. Given that the key variables were measured as ordinal data, Spearman rank correlation analysis was employed to examine the bivariate relationships among perceived institutional support, perceived stigma, and students’ mental health.

Second, a series of multiple regression models were constructed to test the research hypotheses. To ensure the rigor of the results, a set of control variables was included in all models, including gender, age, academic year, residential background before age 18, only-child status, monthly living expenses level, perceived sufficiency of living expenses, psychological counseling experience, and students’ perception of non-institutional support. Academic year was included because students at different stages of university education may differ in their familiarity with campus resources and psychological support services. Given the distinct socioeconomic and infrastructural characteristics of county-level towns in China, childhood residential environment was categorized into three groups: urban areas, rural areas, and county/town areas. Monthly living expenses levels and perceived sufficiency of living expenses were included as indicators related to students’ socioeconomic background. Prior psychological counseling experience and perceived non-institutional support were included to account for previous help-seeking experiences and alternative sources of psychological support. These analyses aimed to examine the direct associations between institutional support and both mental health and perceived stigma.

Finally, the potential indirect association involving perceived stigma was examined using the “sgmediation” command in Stata 18.0. This procedure allowed for the simultaneous estimation of direct and indirect associations within the analytical model. The indirect association was considered statistically significant if the 95% confidence interval did not include zero. This approach provides a statistical assessment of whether perceived stigma may account for part of the association between perceived institutional support and mental health outcomes.

### 3.4 Ethical considerations

This study was reviewed and approved by the Psychological Ethics Committee of Jiangsu University of Technology. All procedures were conducted in accordance with established ethical guidelines for research involving human participants. Participation was voluntary and anonymous.

## 4 Results

### 4.1 Descriptive statistics and correlations

Table 2 presents the participant characteristics and descriptive statistics of the study variables. Panel A reports continuous participant characteristics, Panel B presents categorical participant characteristics, and Panel C provides descriptive statistics and bivariate correlations among the key variables. In the final sample, 45.48% of participants were male and 54.52% were female. The mean age of participants was 19.66 years (SD = 1.24).

**Table 2:** Participant characteristics, descriptive statistics, and correlations among key variables.

Panel A: Continuous participant characteristics		
Variable	Mean	Standard deviation
Age	19.66	1.24

Panel B: Categorical participant characteristics		
Variable	Category	n (%)
Gender	Male	292 (45.48%)
	Female	350 (54.52%)
Academic year	Freshman	272 (42.37%)
	Sophomore	249 (38.79%)
	Junior	106 (16.51%)
	Senior	15 (2.34%)
Residential background before age 18	Urban	162 (25.23%)
	Rural	287 (44.70%)
	County/Town	193 (30.06%)
Only-child status	Yes	200 (31.15%)
	No	442 (68.85%)
Prior psychological counseling experience	Yes	45 (7.01%)
	No	597 (92.99%)
Monthly living expense level	<1,000 RMB	32 (4.98%)
	1,001–1,500 RMB	233 (36.29%)
	1,501–2,000 RMB	249 (38.79%)
	2,001–2,500 RMB	86 (13.40%)
	2,501–3,000 RMB	27 (4.21%)
	3,001–4,000 RMB	8 (1.25%)
	4,001–5,000 RMB	1 (0.16%)
	>5,000 RMB	6 (0.93%)
Perceived sufficiency of living expenses	Very insufficient	6 (0.93%)
	Somewhat insufficient	60 (9.35%)
	Adequate	408 (63.55%)
	Very sufficient	168 (26.17%)

Panel C: Descriptive statistics and bivariate correlations among key variables					
Variables	Mean	Standard deviations	Students’ perceived institutional support	Perceived stigma	Mental health
Students’ perceived institutional support	4.04	0.76	1		
Perceived stigma	2.54	0.62	−0.235*	1	
Mental health	3.49	0.65	0.254*	−0.598*	1

Values in the correlation matrix represent Pearson’s correlation coefficients. Data were collected from the authors’ survey. *p < 0.10, **p < 0.05, ***p < 0.01.

### 4.2 Common method Bias test

Harman’s single-factor test was employed to examine common method bias (CMB). Principal Component Analysis (PCA) was conducted using Stata18.0 on all measurement items for the independent, focal, and outcome variables. The results indicated that five factors had eigenvalues greater than 1. The first principal component explained 36.33% of the variance, which is below the 40% threshold. Therefore, there is no serious common method bias in this study.

### 4.3 Hypothesis testing

This study examined the associations among students’ perceived institutional support, perceived stigma, and mental health outcomes.

#### 4.3.1 The association between students’ perceived institutional support and mental health

As shown in Model 1 (Table 3), after controlling for relevant variables, students’ perceived institutional support were positively associated with mental health. Specifically, higher levels of perceived institutional support were associated with better mental health outcomes. Therefore, H1 was supported.

**Table 3:** Hierarchical regression analysis examining the associations among students’ perceived institutional support, perceived stigma, and mental health.

Variables	Model 1 (MH)	Model 2 (PS)	Model 3 (MH)
IS	0.114*** (0.037) β = 0.130	−0.183*** (0.036) β = −0.226	0.006 (0.030) 0.006
PS	—	—	−0.602***(0.033) β = −0.573
Controls			
Gender	0.100** (0.049) β = 0.077	−0.194*** (0.048) β = −0.157	−0.017 (0.040) β = −0.013
Age	0.048 (0.027) β = 0.091	0.003 (0.026) β = 0.006	0.050** (0.022) β = 0.095
Academic year	−0.039 (0.041) β = −0.048	−0.018 (0.040) β = −0.024	−0.050 (0.033) β = −0.062
Pre-18 residential background	−0.114*** (0.033) β = −0.131	0.072** (0.033) β = 0.087	−0.071***(0.027) β = −0.081
Only-child status	0.005 (0.053) β = 0.003	−0.085 (0.052) β = −0.064	−0.046 (0.043) β = −0.033
Monthly living expenses level	−0.040* (0.024) β = −0.068	0.042* (0.023) β = 0.075	−0.015 (0.019) β = −0.025
Perceived sufficiency of living expenses	0.117 (0.041) β = 0.109	−0.061 (0.040) β = −0.060	0.080** (0.034) β = 0.075
Prior psychological counseling experience	−0.200** (0.096) β = −0.079	−0.091 (0.093) β = −0.038	−0.254*** (0.078) β = −0.100
Perceived non-institutional support	0.238*** (0.047) β = 0.224	−0.075 (0.046) β = −0.074	0.193*** (0.038) β = 0.182
Constant	1.128** (0.522)	3.790* (0.508)	3.411* (0.441)
adj. R^2^	0.146	0.107	0.438
*F* value	11.96***	8.64***	46.5***
*N*	642	642	642

Unstandardized coefficients are reported, with standard errors in parentheses. Standardized coefficients (β) are shown after the corresponding standard errors to facilitate the interpretation of effect sizes. IS, students’ perceived institutional support; MH, mental health; PS, perceived stigma. Data were collected from the authors’ survey. *p < 0.10, **p < 0.05, ***p < 0.01.

#### 4.3.2 The association between students’ perceived institutional support and perceived stigma

As shown in Model 2 (Table 3), after adjusting for relevant control variables, students’ perceived institutional support were significantly and negatively associated with perceived stigma. This finding indicates that students who perceived higher levels of university mental health support tended to report lower levels of perceived stigma. Therefore, H2 was supported.

#### 4.3.3 The potential indirect association through perceived stigma

The results of Model 3 (Table 3) indicated that, when students’ perceived institutional support and perceived stigma were entered into the regression model simultaneously, perceived stigma was significantly negatively associated with mental health outcomes.

To further examine the significance of the indirect association, a bootstrapping procedure with 5,000 resamples was conducted. The results showed that the estimated indirect association was 0.110, with a bias-corrected 95% bootstrap confidence interval of [0.061, 0.162]. Because the confidence interval did not include zero, the indirect association was statistically significant (see Table 4). These findings suggest that perceived stigma may partially account for the observed association between students’ perceived institutional support and mental health outcomes. Therefore, H3 was supported.

**Table 4:** Bootstrap analysis of the potential indirect association between support perceptions and mental health outcomes through perceived stigma.

Association type	Estimate	Bootstrap SE	LLCI (95%)	ULCI (95%)
Indirect association	0.110	0.025	0.061	0.162
Direct association	0.004	0.032	−0.064	0.064
Total association	0.114	0.045	0.022	0.199

Bootstrap estimates were based on 5,000 resamples. LLCI and ULCI represent the lower and upper limits of the 95% confidence interval, respectively. The indirect association was considered statistically significant when the 95% confidence interval did not include zero. The proportion of the indirect association was calculated as the ratio of the indirect association to the total association. Data were collected from the authors’ survey.

#### 4.3.4 Magnitude of observed associations

To evaluate the magnitude of the observed associations, standardized coefficients were examined. The results indicated that the association between students’ perceived institutional support and mental health was small in magnitude (β = 0.130). The association between students’ perceived institutional support and perceived stigma was small to moderate (β = −0.224). In contrast, perceived stigma showed a relatively stronger negative association with mental health outcomes (β = −0.573). These findings suggest that stigma-related perceptions may represent an important factor associated with students’ mental health outcomes, while institutional support perceptions may be related to mental health partly through stigma-related processes.

### 4.4 Robustness checks

To accounts for potential unobserved regional heterogeneity that may influence students’ perceived institutional support, perceived stigma, and mental health outcomes, additional analyses were conducted by incorporating county-level fixed effects into the regression models.

As shown in Table 5, after including province fixed effects, the associations between students’ perceived institutional support, perceived stigma, and mental health remained largely consistent with the main findings. Specifically, students’ perceived institutional support remained positively associated with mental health (Model 4) and negatively associated with perceived stigma (Model 5). Moreover, perceived stigma remained negatively associated with mental health outcomes after the inclusion of province fixed effects (Model 6). These findings suggest that the observed associations were not substantially affected by regional differences.

**Table 5:** Robustness checks with provincial fixed effects.

Variables	Model 4 (MH)	Model 5 (PS)	Model 6 (MH)
IS	0.115*** (0.037)	−0.187*** (0.036)	0.003 (0.030)
PS			−0.597*** (0.033)
Controls			
Gender	0.113** (0.049)	−0.206*** (0.048)	−0.010 (0.041)
Age	0.042 (0.027)	0.009 (0.026)	0.047** (0.022)
Academic year	−0.063 (0.042)	−0.002 (0.041)	−0.064* (0.034)
Pre-18 residential background	−0.111*** (0.033)	0.072** (0.033)	−0.068** (0.027)
Only-child status	0.019 (0.054)	−0.093* (0.052)	−0.036 (0.044)
Monthly living expenses level	−0.035 (0.024)	0.036 (0.023)	−0.014 (0.019)
Perceived sufficiency of living expenses	0.099** (0.042)	−0.052 (0.041)	0.068** (0.034)
Prior psychological counseling experience	−0.214** (0.095)	−0.077 (0.093)	−0.260*** (0.077)
Perceived non-institutional support	0.238*** (0.047)	−0.078* (0.046)	0.191*** (0.038)
Constant	1.536*** (0.561)	3.654*** (0.549)	3.719*** (0 0.472)
Province fixed effects	Yes	Yes	Yes
adj. R^2^	0.154	0.101	0.442
*N*	642	642	642

IS, students’ perceived institutional support; MH, mental health; PS, perceived stigma. Data were collected from the authors’ survey. Standard errors in parentheses. Data were collected from the authors’ survey. *p < 0.10, **p < 0.05, ***p < 0.01.

## 5 Discussion

### 5.1 Main findings and interpretation

This study examined the associations among student’s perceived institutional support, perceived stigma, and mental health within the context of high awareness but low utilization of university counseling services in China. Rather than focusing solely on whether students access counseling services, this study explored whether institutional support may carry psychological significance through students’ perceptions of the support resources. Three main findings emerged.

First, student’s perceived institutional support were positively associated with mental health after adjusting for demographic characteristics and other sources of support. This finding extends previous research on social support by suggesting that institutional environments may represent an additional source of psychological resources beyond interpersonal relationships. Prior studies have consistently demonstrated that perceived social support is associated with better psychological outcomes and may be a stronger predictor of well-being than objective support availability or actual support use (Wills and Shinar, 2000; Haber et al., 2007). Consistent with this perspective, the present findings suggest that students’ perceptions of available institutional resources may have psychological relevance even when actual counseling utilization remains limited.

Second, perceived stigma showed a significant negative association with mental health and statistically accounted for part of the observed association between student’s perceived institutional support and mental health. This finding is consistent with extensive international research indicating that stigma is closely related to psychological distress, negative attitudes toward help-seeking, and reluctance to disclose mental health concerns (Vogel et al., 2007; Lannin et al., 2016). Previous studies have often conceptualized stigma primarily as an individual-level barrier that prevents students from seeking professional support. The present study extends this perspective by suggesting that stigma-related perceptions may also be considered within broader institutional and social contexts.

The observed association between perceived institutional support and perceived stigma may reflect the potential role of organizational signals in shaping students’ interpretations of mental health-related norms. Universities that formally acknowledge psychological difficulties and provide visible support resources may be perceived by students as environments where mental health concerns are more recognized and socially acceptable.

This interpretation may be particularly relevant in the Chinese higher education context, where cultural norms regarding social evaluation, interpersonal harmony, and collective expectations may influence how students perceive and respond to psychological difficulties. In collectivistic cultural contexts, individuals may place greater emphasis on maintaining positive social relationships and avoiding negative evaluations from others, which may increase concerns about disclosing psychological difficulties or seeking professional support (Yang et al., 2007; Xu et al., 2017). Under such circumstances, institutional endorsement of mental health support may carry symbolic meaning beyond the provision of services, as students may interpret such support as an indication that psychological difficulties are legitimate and acceptable concerns within the university community.

However, given the cross-sectional nature of the data, this interpretation should be understood as a potential explanation of the observed associations rather than evidence of a causal process.

Beyond interpreting the observed indirect association, these findings also provide insight into why high awareness of counseling services does not necessarily result in high utilization. The persistence of low counseling utilization despite high awareness suggests that awareness alone may be insufficient to translate into actual help-seeking behaviors. The gap between awareness and utilization may reflect a transition problem from cognitive recognition to behavioral engagement. Although awareness indicates that students recognize the existence of available services, actual utilization requires additional psychological readiness, perceived safety, and social acceptance of help-seeking. Previous research has suggested that awareness of mental health services represents only an initial step in the help-seeking process, whereas actual service use is shaped by multiple psychological and social factors, including self-stigma, concerns about social evaluation, perceived confidentiality, and attitudes toward professional assistance (Rickwood et al., 2005; Vogel et al., 2007; Gulliver et al., 2010).

In particular, self-stigma, concerns about being judged by others, and fear of potential discrimination may discourage students from seeking professional support even when services are available (Corrigan et al., 2006; Vogel et al., 2007). In the Chinese cultural context, where social evaluation, interpersonal harmony, and the avoidance of negative judgments may be particularly salient, students may be reluctant to disclose psychological difficulties or seek formal support (Yang et al., 2007; Xu et al., 2017). Moreover, some students may normalize psychological distress as an expected consequence of academic pressure and therefore perceive professional assistance as unnecessary.

These factors suggest that improving awareness and accessibility of counseling services alone may not be sufficient to increase utilization. Instead, reducing psychological and social barriers to help-seeking may be equally important. In this regard, institutional support may play a broader role beyond providing counseling resources by shaping students’ perceptions of whether seeking psychological help is acceptable, safe, and socially supported within the university environment.

Third, consistent with previous social support literature, perceived non-institutional support remained positively associated with mental health outcomes. This finding reinforces the importance of interpersonal relationships, including support from family members and friends, as established psychological resources. At the same time, the independent association of perceived institutional support suggests that institutional and interpersonal support may represent complementary rather than interchangeable forms of support. While interpersonal relationships provide individualized emotional and practical assistance, institutional support may reflect students’ broader perceptions of whether their educational environment recognizes and accommodates psychological needs.

It is important to interpret the magnitude of these associations beyond statistical significance. Although the associations between perceived institutional support, perceived stigma, and mental health were statistically significant, their effect sizes were relatively modest. Given the relatively large sample size, statistically significant findings may be detected even when the magnitude of associations is limited. Therefore, these results should not be interpreted as indicating that perceived institutional support is a dominant determinant of students’ mental health or that changes in institutional support alone would lead to substantial improvements in psychological outcomes.

Rather, the findings suggest that perceived institutional support may represent one of multiple contextual factors associated with students’ mental health. Its practical relevance may lie in its potential relevance for understanding and evaluating supportive and less stigmatizing institutional environments, where even modest psychological benefits may become meaningful when considered alongside other individual and contextual determinants. Future research should further examine whether strengthening institutional support produces clinically meaningful changes in mental health outcomes through longitudinal or intervention-based designs.

### 5.2 International perspectives and theoretical implications

The findings provide a complementary perspective to previous research from North America and Europe, where university mental health initiatives have increasingly emphasized improving service accessibility, expanding counseling availability, and reducing barriers to professional help-seeking. However, previous studies have consistently shown that increased awareness and availability of counseling services do not necessarily translate into actual utilization among college students (Eisenberg et al., 2007; Lipson et al., 2019). Research in these contexts has largely focused on behavioral barriers to service use, including negative attitudes toward help-seeking, perceived stigma, concerns about confidentiality, and reluctance to disclose psychological difficulties. Beyond actual service utilization, the present study complements this literature by highlighting that students’ perceptions of university mental health support may represent an additional dimension through which institutional environments are evaluated.

Similar challenges have also been observed in other Asian contexts, where mental health help-seeking among university students may be shaped by stigma, concerns about social evaluation, and reluctance to disclose psychological difficulties. Studies conducted in Japan have highlighted the persistent influence of mental health stigma on public attitudes and help-seeking behaviors, suggesting that concerns about negative social evaluation may discourage individuals from acknowledging psychological problems or seeking professional support (Ando et al., 2013; Masuda et al., 2005). Similarly, research among Korean university students has indicated that mental health literacy alone may not be sufficient to promote help-seeking, as stigma and cultural barriers continue to influence students’ willingness to access psychological services (Kim, 2021). These findings suggest that, across several Asian societies, barriers to mental health service utilization may extend beyond service availability and involve broader social and cultural interpretations of psychological distress (Lauber and Rössler, 2007).

Within this broader Asian context, the Chinese higher education environment provides a particularly relevant setting for examining the role of institutional support. Chinese universities often represent important institutional environments where students access psychological resources and form perceptions regarding the legitimacy and acceptability of mental health concerns. Given the influence of academic competition, concerns about social evaluation, and the importance placed on interpersonal harmony, students may be more cautious about disclosing psychological difficulties or seeking professional assistance (Yang et al., 2007, 2014; Xu et al., 2017). Under such circumstances, institutional recognition of mental health concerns may carry significance beyond the provision of counseling services. Under such circumstances, institutional recognition of mental health concerns may carry significance beyond the provision of counseling services. Visible institutional support may be interpreted by students as communicating that psychological difficulties are legitimate, acceptable, and worthy of attention. This interpretation is consistent with perspectives emphasizing that social and organizational environments may be associated with stigma-related experiences through their influence on norms, expectations, and the social meanings attached to mental health problems (Link and Phelan, 2001; Hatzenbuehler, 2016).

These international comparisons should also be interpreted in light of methodological differences across studies. Previous research has often examined university mental health support from a service utilization perspective, focusing on outcomes such as counseling uptake, help-seeking intentions, or willingness to seek professional assistance. In contrast, the present study focused on perceived institutional support and examined its associations with perceived stigma and mental health outcomes. Therefore, the current findings complement rather than replace previous research by suggesting that the role of university mental health support may extend beyond facilitating service use and may also involve students’ perceptions of the broader institutional environment.

Differences in measurement approaches should also be considered when interpreting cross-study comparisons. Many previous studies have assessed stigma through specific dimensions, such as self-stigma or public stigma, whereas the present study integrated these dimensions into an adapted measure to capture broader stigma-related perceptions among students. Similarly, perceived institutional support in the current study was developed to assess students’ perceptions of support from university-based psychological and professional resources rather than objective service availability or actual utilization. These conceptual and measurement differences may contribute to variations in findings across studies.

Finally, differences in research design further limit direct comparisons across studies. Many previous studies have adopted longitudinal or experimental designs to examine changes in help-seeking attitudes or mental health outcomes, whereas the present cross-sectional study identifies associations among perceived institutional support, stigma, and mental health at a single time point. Therefore, the findings should be interpreted as evidence of theoretically consistent associations rather than causal mechanisms.

Taken together, these findings contribute to the literature in three ways. First, Beyond a utilization-based perspective, the present study suggests that university mental health support may also be evaluated through students’ perceptions of institutional recognition, psychological safety, and social acceptance. This perspective extends existing research by considering institutional support as a perceived contextual resource rather than solely as a service-delivery mechanism.

In addition, this study contributes to stigma research by emphasizing that stigma-related perceptions may also be understood within institutional contexts. While traditional stigma research has largely focused on individual attitudes and interpersonal judgments (Goffman, 1963), recent perspectives have highlighted the relevance of structural and organizational environments (Hatzenbuehler, 2016). The present study extends this perspective by examining perceived stigma in relation to students’ perceptions of university support environments.

Finally, the measurement approach used in this study extends previous stigma research by integrating two theoretically distinct but related dimensions of mental health stigma: self-stigma and perceived public stigma. Previous studies have typically examined these dimensions separately, focusing either on individuals’ internalized negative beliefs about seeking psychological help or their perceptions of societal attitudes toward help-seeking (Vogel et al., 2007; Barney et al., 2006). In the present study, these dimensions were conceptualized as complementary components of perceived stigma within the university context. After item-level evaluation and confirmatory factor analysis, three items with insufficient item discrimination were removed, resulting in a refined 12-item scale with satisfactory reliability and construct validity. This adaptation suggests that the revised measure retains the conceptual distinction between self-related and socially perceived stigma while improving its applicability among Chinese college students.

### 5.3 Practical implications

Within the context of Chinese higher education, the findings may have several practical implications for the development and evaluation of university mental health support systems. First, the results suggest that evaluating institutional mental health services solely based on utilization rates may provide an incomplete picture of their value. Although counseling utilization remains an important indicator of service accessibility, the psychological significance of institutional support may also exist through students’ perceptions of availability, acceptance, and legitimacy. Therefore, universities may consider incorporating indicators related to students’ awareness, perceived accessibility, and psychological acceptance of support resources when assessing campus mental health initiatives.

Second, the findings highlight the potential importance of institutional communication and normative signals in creating a supportive mental health environment. Universities may strengthen mental health promotion through official communication channels, faculty training, and student support programs that convey the message that psychological difficulties are common and legitimate aspects of student experiences. Such efforts should not be limited to encouraging students to seek counseling services but may also focus on fostering a campus climate in which mental health discussions are perceived as acceptable and non-stigmatizing.

Third, the findings indicate that institutional support should be viewed as complementary to, rather than a replacement for, interpersonal support networks. While family and peer relationships remain important sources of psychological support, universities occupy a unique position as formal organizations responsible for creating inclusive and accessible environments. Integrating institutional resources with peer support programs, faculty guidance, and existing student services may provide a more comprehensive approach to addressing college students’ mental health needs.

Importantly, these implications should be interpreted within the context of Chinese higher education. The extent to which institutional support functions similarly in other educational systems may depend on cultural norms, organizational structures, and the accessibility of mental health services. Future comparative research is therefore needed to examine whether these patterns can be generalized across different institutional and cultural contexts.

### 5.4 Limitations and future research

Several limitations should be acknowledged. First, although mediation analysis provides a useful framework for examining potential mechanisms, the cross-sectional nature of the present study requires careful interpretation of the observed indirect association. Mediation models generally imply a temporal sequence in which the predictor precedes the mediator and the mediator precedes the outcome. However, because perceived institutional support, perceived stigma, and mental health were measured simultaneously, the present study cannot establish whether changes in institutional support perceptions precede changes in stigma or whether stigma precedes changes in mental health. Therefore, the identified indirect pathway should be interpreted as a theoretically consistent association rather than definitive evidence of a causal mechanism.

Alternative explanations should also be considered. For example, students with better psychological well-being may evaluate their institutional environments more positively and consequently report higher levels of perceived institutional support, suggesting a possible reverse direction of association. Similarly, although perceived stigma was conceptualized as a potential explanatory pathway in the present study, it may also reflect existing psychological difficulties, as students experiencing poorer mental health may become more sensitive to social evaluation and perceive greater stigma. Moreover, unmeasured individual and contextual factors, such as physical activity, obesity, previous psychiatric disorders, medication use, alcohol and substance use, family functioning, and prior sleep quality, may simultaneously influence perceived institutional support, perceived stigma, and mental health outcomes. Future longitudinal and experimental studies are needed to clarify the temporal ordering and mechanisms underlying these relationships.

Second, although several demographic and psychosocial covariates were controlled for in the present study, some potentially important individual and contextual factors were not assessed, including physical activity, obesity, previous psychiatric disorders, medication use, alcohol and substance use, family functioning, and prior sleep quality. Therefore, residual confounding cannot be completely excluded. If these unmeasured factors are associated with both perceived institutional support or perceived stigma and mental health, the observed associations may have been overestimated. Because these variables were not available in the current dataset, additional sensitivity analyses incorporating these potential confounders could not be conducted. Future studies should include more comprehensive assessments of behavioral, clinical, and contextual factors and further evaluate whether the observed associations remain robust after accounting for these additional variables.

Third, the sample was drawn from Chinese higher education institutions and included a relatively high proportion of vocational college students. Although this sample provides valuable insights into the mental health context of Chinese college students, caution should be exercised when generalizing the findings to other student populations, educational systems, or cultural settings. Future research should include more diverse institutional types and conduct cross-cultural comparisons to examine whether the observed associations vary across educational environments.

Finally, the measurement of institutional support in this study focused on students’ perceptions of available university mental health resources and formal support roles. While this approach captures an important psychological dimension of institutional support, future research may further examine multiple dimensions of institutional support, including organizational commitment, service quality, confidentiality perceptions, and actual institutional practices. Such multidimensional assessments may provide a more comprehensive understanding of how university mental health environments are experienced by students.

## 6 Conclusion

This study provides evidence that, within the Chinese university context, perceived institutional support is associated with mental health even when utilization rates of counseling services are relatively low. The findings suggest that perceived stigma may partially account for the observed association between perceived institutional support and mental health. However, given the cross-sectional design, these findings should be interpreted as an indirect association consistent with the proposed theoretical framework rather than evidence of a causal mechanism. Whether similar patterns of association exist in other educational and cultural contexts requires further empirical validation. Nevertheless, the present study highlights the importance of considering both the availability of mental health services and students’ perceptions of institutional support when understanding campus mental health environments.

## Data Availability

The original contributions presented in the study are included in the article/Supplementary material, further inquiries can be directed to the corresponding authors.
