# Supplementary material for: High awareness but low utilization: How perceived institutional support shapes college students’ mental health through stigma?
Source: Front Psychol. 2026 Sep 17;17:1897669. doi: 10.3389/fpsyg.2026.1897669 (PMC13627291; doi:10.3389/fpsyg.2026.1897669)
Supplement: Supplementary file 1 [file Data_Sheet_1.PDF]

Table S1. Item performance and standardized factor loadings of the perceived stigma scale

Dimension	Item	Corrected item-total correlation	Standardized factor loading	Decision
Self-stigma	Self1	0.550	0.679	Retained
	Self2	-0.033	-0.100	Removed
	Self3	0.644	0.749	Retained
	Self4	0.573	0.656	Retained
	Self5	0.258	0.083	Removed
	Self6	0.135	-0.044	Removed
	Self7	0.607	0.848	Retained
	Self8	0.745	0.696	Retained
	Self9	0.641	0.864	Retained
	Self10	0.771	0.620	Retained
Public stigma	Public1	0.754	0.835	Retained
	Public2	0.796	0.902	Retained
	Public3	0.827	0.906	Retained
	Public4	0.568	0.553	Retained
	Public5	0.674	0.671	Retained

Note: Items with corrected item-total correlations below 0.30 were considered for removal. Standardized factor loadings were obtained from confirmatory factor analysis (CFA).

Table S2. Fit indices of confirmatory factor analysis models

Model	χ^2	df	χ^2/df	CFI	TLI	RMSEA	SRMR
Initial 15-item two-factor model	461.87	89	5.19	0.757	0.713	0.149	0.132
Revised 12-item two-factor model	126.67	53	2.39	0.920	0.908	0.046	0.078
